# Neuroprotection Exerted by Netrin-1 and Kinesin Motor KIF1A in Secondary Brain Injury following Experimental Intracerebral Hemorrhage in Rats

**DOI:** 10.3389/fncel.2017.00432

**Published:** 2018-01-11

**Authors:** Jun Wang, Weiwei Zhai, Zhengquan Yu, Liang Sun, Haiying Li, Haitao Shen, Xiang Li, Chunfeng Liu, Gang Chen

**Affiliations:** ^1^Department of Neurology, The Second Affiliated Hospital of Soochow University, Suzhou, China; ^2^Department of Neurology, Yancheng City No.1 People's Hospital, Yancheng, China; ^3^Department of Neurosurgery and Brain and Nerve Research Laboratory, The First Affiliated Hospital of Soochow University, Suzhou, China; ^4^Laboratory of Aging and Nervous Diseases, Institute of Neuroscience, Soochow University, Suzhou, China

**Keywords:** intracerebral hemorrhage, secondary brain injury, apoptosis, netrin-1, DCC, UNC5H2, KIF1A

## Abstract

Binding of extracellular netrin-1 to its receptors, deleted in colorectal cancer (DCC) and uncoordinated gene 5H2 (UNC5H2), inhibits apoptosis mediated by these receptors. A neuron-specific kinesin motor protein, KIF1A, has been shown to participate in netrin-1 secretion. This study aimed to identify the roles of netrin-1 and KIF1A in secondary brain injury after intracerebral hemorrhage (ICH) and the potential mechanisms. An autologous blood ICH model was established in adult male Sprague-Dawley rats, and cultured neurons were exposed to OxyHb to mimic ICH conditions *in vitro*. Mouse recombinant netrin-1, expression vectors encoding KIF1A, and KIF1A-specific siRNAs were administered intracerebroventricularly. After ICH, protein levels of netrin-1, DCC, and UNC5H2 increased, while protein levels of KIF1A decreased. Levels of UNC5H2 and DCC bound to netrin-1 increased after ICH but were significantly lower than the increase in total amount of protein. Administration of recombinant netrin-1 attenuated neuronal apoptosis and degeneration in ICH rats. Moreover, KIF1A overexpression increased concentrations of netrin-1 in cerebrospinal fluid and cell culture supernatant and exerted neuroprotective effects via netrin-1 and its receptor pathways. KIF1A plays a critical role in netrin-1 secretion by neurons. An increase in protein levels of netrin-1 may be a neuroprotective strategy after ICH. However, this process is almost completely abolished by ICH-induced loss of KIF1A. An exogenous increase of KIF1A may be a potential strategy for neuroprotection via the netrin-1 pathway.

## Introduction

Intracerebral hemorrhage (ICH) is a common and often fatal type of stroke with high morbidity and mortality, and it frequently leads to long-lasting neurological dysfunctions (Bonatti et al., 2016; Ji et al., 2016; Lan et al., 2017). Spontaneous ICH has been reported to account for ~4–14% of all strokes, with a higher reported incidence in Asian countries when compared with the West (Broderick et al., 1999; Onwuchewa et al., 2009). Only 20% of patients are independent within 6 months, and about 32–50% die within the first month after ICH (Huang et al., 2008). In addition to primary brain injury caused by direct mechanical injury of hemorrhage, ICH also leads to secondary brain injury (SBI), which has been shown to contribute to neurological deterioration (Mittal and LacKamp, 2016; Li et al., 2017).

Netrin-1 has been proposed to act as a diffusible chemotropic factor that attracts or repels axons, depending on which netrin receptor is expressed on the individual axon (Colamarino and Tessier-Lavigne, 1995; Charron et al., 2003). Recently, it was reported that netrin-1 supplied by neural progenitors promotes axon guidance in the spinal cord (Varadarajan et al., 2017). Netrin-1 and its receptors, deleted in colorectal cancer (DCC) and uncoordinated gene 5H2 (UNC5H2), are highly expressed in the central nervous system from embryonic development through adulthood (Serafini et al., 1996; Strähle et al., 1997; Sim et al., 1999; Manitt et al., 2006; Birey and Aguirre, 2015). Known as dependence receptors, DCC and UNC5H2 trigger either survival or apoptotic signals depending on the presence or absence of their ligand, netrin-1 (Mehlen and Mazelin, 2003; Delcros and Mehlen, 2013; Mehlen and Tauszig-Delamasure, 2014). Many studies have shown an important role of netrin-1 and its receptors in diseases of the central nervous system (Arakawa, 2004; Lin and Isacson, 2006; Wu et al., 2008; Podjaski et al., 2015; Lu et al., 2016). Although netrin-1 has been previously investigated in middle cerebral artery occlusion and traumatic brain injury models (Wu et al., 2008; Wen et al., 2014), there is no information regarding the role of netrin-1 in ICH-induced SBI.

KIF1A, which is a neuron-specific kinesin-3 family motor protein, is a globular, monomeric molecule and, at about 1.2 microns/s, has the fastest reported anterograde motor activity in axonal transport of synaptic vesicle precursors (Okada et al., 1995). KIF1A is thought to bind its cargo in the cell body of the neuron, transport the cargo along microtubule tracks to synapses, and release the cargo upon reaching the synapse (Hirokawa et al., 2009). It has been reported that KIF1A transports vesicles containing netrin-1 from the neuronal cell body to the axon and is involved in synaptic secretion of netrin-1 (Ogura et al., 2012).

Therefore, this study investigated the role of netrin-1 in ICH-induced SBI and the underlying mechanisms, especially its secretion and receptors.

## Materials and methods

### Recombinant protein and antibodies

Recombinant mouse netrin-1 (1109-N1-025) was purchased from R&D systems (Minneapolis, MN, USA). Anti-netrin-1 antibodies (ab126729 and ab122903), anti-DCC antibody (ab125280), anti-UNC5H2 antibody (ab189914), anti-KIF1A antibody (ab180153), and Ms mAb to NeuN (ab104224) were purchased from Abcam (Cambridge, MA, USA). Cleaved caspase-3 (Asp175) antibody (9661) was purchase from Cell Signaling Technology (Danvers, MA, USA). Normal rabbit IgG (sc-2027) and anti-β-tubulin antibody (sc-365791) were purchased from Santa Cruz Biotechnology (Santa Cruz, CA, USA). An ELISA kit for netrin-1 was purchased from Cloud-Clone (SEB827Ra, Houston, TX, USA). Secondary antibodies for immunofluorescence microscopy, including Alexa Fluor-488 donkey anti-rabbit IgG antibody (A21206), Alexa Fluor-555 donkey anti-mouse IgG antibody (A31570), Alexa Fluor-488 donkey anti-goat IgG antibody (A11055), and Alexa Fluor-647 donkey anti-rabbit IgG antibody (ab150075) were obtained from Invitrogen (Carlsbad, CA, USA). Secondary antibodies for western blot analysis, including anti-rabbit IgG horseradish peroxidase (HRP)-linked antibody (7074) and anti-mouse IgG HRP-linked antibody (7076) were purchased from Cell Signaling Technology.

### Animals

All experiments and procedures were approved by the Ethics Committee of the First Affiliated Hospital of Soochow University and in accordance with the guidelines of the National Institutes of Health on the care and use of laboratory animals. Adult male Sprague-Dawley rats weighing 280–320 g and pregnant Sprague-Dawley rats of gestation 17-days were purchased from the Animal Center of the Chinese Academy of Sciences, Shanghai, China. The rats were housed in temperature- and humidity-controlled animal quarters with a 12-h light/dark cycle.

### Establishment of the experimental ICH model in rats

**Figure 1:**
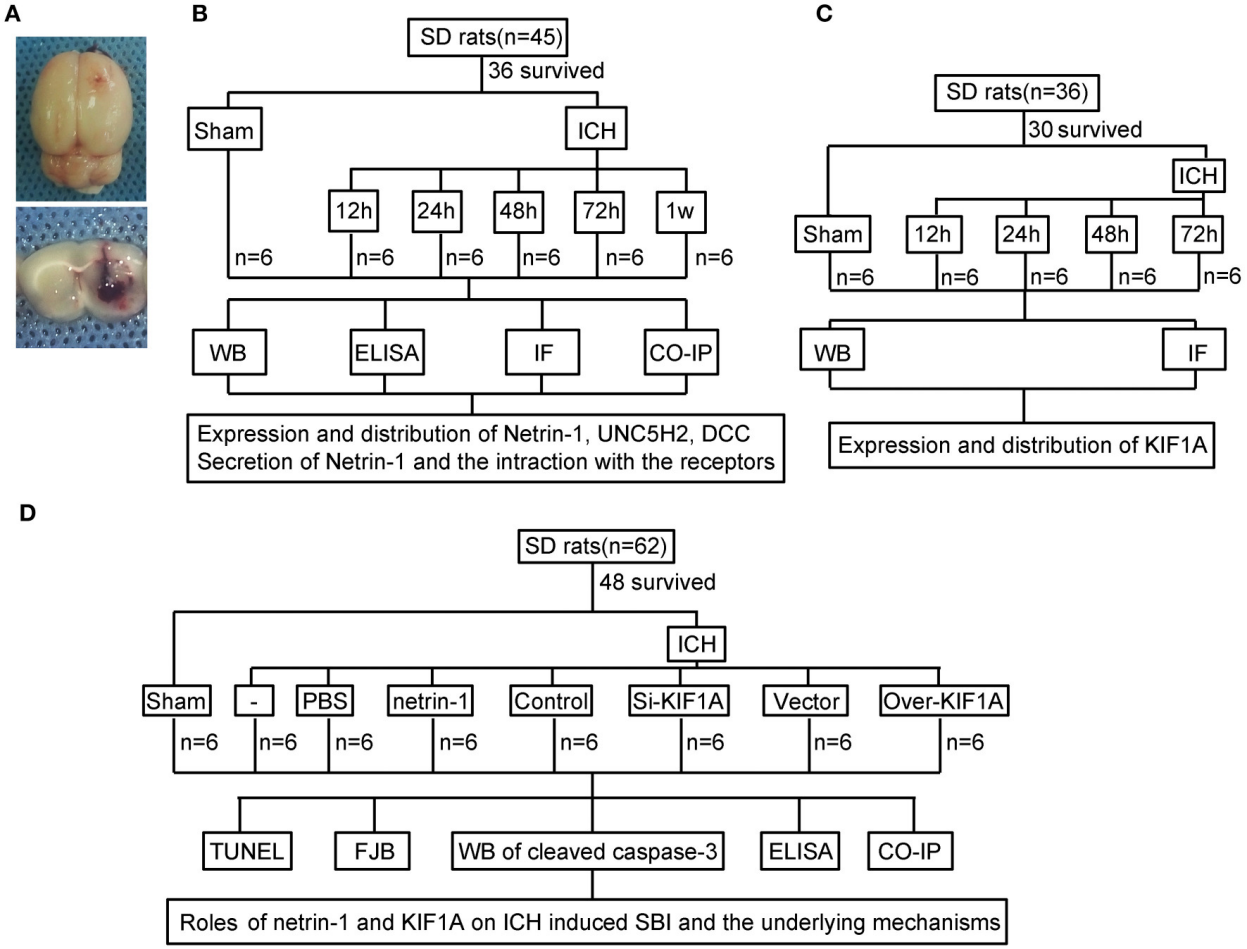
Experimental design.**(A)** Representative gross brain morphology and representative coronal slices of the rat ICH model. **(B)** Experimental design to evaluate expression, secretion, and localization of netrin-1, expression and localization of DCC and UNC5H2, and interactions between ligand and receptors following ICH. **(C)** Experiment designed to investigate changes in levels of motor protein, KIF1A, after ICH. **(D)** Experiment designed to elucidate the roles of netrin-1 and KIF1A in ICH-induced SBI and potential mechanisms.

An experimental rat ICH model was induced by autologous whole blood injection into the basal ganglia. The position of the basal ganglia was 3.5 mm lateral to the midline, 0.2 mm posterior to bregma, and 5.5 mm ventral to the cortical surface. After the microinjector was in position, autologous whole blood (100 μL) was injected over 5 min, and the needle was left in the brain for 5 min. Bone wax was used to block the burr hole to prevent loss of cerebrospinal fluid (CSF) and blood from the midline vessels. Finally, the scalp was sutured, and the rat was returned to its cage where it had free access to food and water. Representative brain slices from the ICH model are shown in Figure 1A.

### Primary neuronal cultures and establishment of an experimental ICH model *in vitro*

As described previously (Shen et al., 2015), the cortices of 17-days rat embryos were used for primary neuron collection. After blood vessels and the meninges were removed, the brains were digested with trypsin for 5 min. Next, the brain suspension was centrifuged at 500 × g for 5 min. Finally, the collected neurons were inoculated into neurobasal medium (GIBCO, Carlsbad, CA, USA) and maintained in a 5% CO_2_ atmospheric incubator at 37°C. The medium was renewed every 2 days for 2 weeks.

As reported previously (Zhai et al., 2016), the neurons were treated with OxyHb (10 μM) to mimic ICH conditions *in vitro*.

### Experiment grouping

As shown in Figure 1B, 36 rats (45 rats were used, 36 rats survived surgery) were randomly assigned to six groups (*n* = 6 per group): a sham group and five model groups assessed at 12, 24, 48, 72, and 1 week after ICH. Brain tissues and CSF of the six rats in each group were collected at the indicated time points and used for western blot analysis, immunofluorescence analysis, enzyme-linked immunosorbent assay (ELISA), and co-immunoprecipitation (CO-IP) to evaluate effects of ICH insults on protein levels of netrin-1 and its receptors in the peri-hematoma cortex. *In vitro*, corresponding experiments were performed in cultured neurons exposed to OxyHb.

In addition, 30 rats (36 rats were used, 30 rats survived surgery) were randomly assigned to five groups (*n* = 6 per group): a sham group and four model groups assessed at 12, 24, 48, and 72 h after ICH. Brain tissues of the six rats in each group were collected at the indicated time points and used for western blot and immunofluorescence analysis to evaluate effects of ICH insults on protein levels and distribution of KIF1A in the peri-hematoma cortex (Figure 1C).

Finally, 48 rats (62 rats were used, 48 rats survived surgery) were randomly assigned to eight groups (*n* = 6 per group): sham group, ICH group, ICH + PBS group, ICH + mouse recombinant netrin-1 group, ICH + control siRNA group, ICH + KIF1A-siRNA group, ICH + vector group, and ICH + KIF1A-overexpresssion group. Transfection of plasmids and siRNAs were performed 48 h before surgery, and injection of mouse recombinant netrin-1 was done 6 h after ICH onset. Brain tissues and CSF of the six rats in each group were collected at 48 h after ICH onset and used to evaluate the roles of netrin1 and KIF1A in ICH-induced SBI and the underlying mechanisms (Figure 1D). *In vitro*, corresponding experiments were performed on cultured neurons exposed to OxyHb.

### Mouse recombinant netrin-1 treatment

Recombinant mouse netrin-1 (200 ng in 2 μL PBS) was prepared according to the manufacturer's instructions and administered via intracerebroventricular injection as previously described (Wu et al., 2008; Zhai et al., 2016).

### Transfection

To overexpress KIF1A and knockdown the KIF1A gene, a specific expression plasmid and siRNAs against rat KIF1A were obtained from GenScript (Nanjing, Jiangsu Province, China). The construct of the expression plasmid was confirmed by DNA sequencing. Interference efficiency of three different siRNAs was tested, and the most efficient one (siRNA-II) was used in this study. The three target sequences for siRNA design are shown below.

- CCTGATGCGGGAAATGTAT
- GCATCCATTCCCACCTCTT
- GCCCAATCGTCTCCAAGAA

*In vivo* plasmid transfection in the rat brain was performed according to the manufacturer's instructions for Entranster-*in vivo* DNA transfection reagent (Engreen, 18668-11-2, Engreen Biosystem Co. Ltd., Beijing, China). First, 10 μL Entranster-*in vivo* DNA transfection reagent was added to 5 μL plasmid (1 μg/μL) or 5 μL empty vector. The solution was mixed for 15 min at room temperature. Next, 15 μL of the mixture was injected intracerebroventricularly at 48 h before ICH.

According to the manufacturer's instructions for Entranster-*in vivo* RNA transfection reagent (Engreen, 18668-11-1), the transfection complex of siRNA was prepared as follows. Briefly, 5 nmol KIF1A siRNA or 5 nmol scramble siRNA was dissolved in 66.5 μL DEPC RNase-free water. Then, 5 μL Entranster-*in vivo* RNA transfection reagent and 5 μL normal saline were added to 10 μL siRNA or 10 μL scramble siRNA. The solution was mixed for 15 min at room temperature. Finally, 20 μL of the mixture was injected intracerebroventricularly at 48 h before ICH.

### Western blot

The peri-hematoma cortex was sampled 1 mm away from the hematoma to avoid potential red blood cell contamination. For total protein extraction, the peri-hematoma cortex or cultured neurons were lysed mechanically in ice-cold RIPA lysis buffer (Beyotime, Shanghai, China). The lysates were then centrifuged at 12,000 × g for 20 min at 4°C, and protein concentration was measured by the bicinchoninic acid (BCA) method using an enhanced BCA protein assay kit (Beyotime). Next, protein samples (50 μg/lane for tissue samples, 25 μg/lane for cell samples) were separated by SDS-PAGE and electrotransferred onto polyvinylidene fluoride membranes (Millipore, Bedford, MA, USA). The membranes were blocked with 5% non-fat milk for 1 h at room temperature and then incubated with primary antibodies followed by the appropriate HRP-conjugated secondary antibodies. β-tubulin was used as a loading control. Finally, protein bands were visualized using SuperSignal West Femto Maximum Sensitivity Substrate (ThermoFisher Scientific, Waltham, MA, USA). The relative quantity of proteins was analyzed using Image J and normalized to that of loading controls.

### Immunofluorescence staining

Double immunofluorescence staining was performed as previously described (Wang et al., 2017). For *in vivo* experiments, the brain samples were fixed in 4% paraformaldehyde, embedded in paraffin, and cut into 4-μm sections, which were dewaxed immediately before immunofluorescence staining. For *in vitro* experiments, cultured neurons were fixed in 4% paraformaldehyde. Next, the sections or cultured neurons were incubated with primary antibodies and appropriate secondary antibodies. Finally, sections and cells were observed using a fluorescence microscope (Olympus BX50/BX-FLA/DP70; Olympus Co., Tokyo, Japan) or a laser scanning confocal microscope (Zeiss LSM 880, Carl Zeiss AG, Oberkochen, Germany). At least six random sections from each sample were examined, and representative results are shown. Quantitative analysis was performed by an observer who was blind to the experimental groups.

### ELISA

CSF concentrations of netrin-1 were determined by ELISA using the rat netrin-1 kit. This assay was performed according to the manufacturer's instructions, and the data are expressed relative to a standard curve for netrin-1.

### CO-IP analysis

We performed immunoprecipitation analysis as described previously (Shen et al., 2015). First, the protein sample was incubated with normal rabbit IgG (negative control) or specific antibodies for 1 h at 4°C with agitation. Next, 50 μL protein A+G agarose beads (Beyotime) was added to each immune complex, and the lysate-bead mixture was incubated overnight with rotary agitation at 4°C. Finally, immunoblotting was performed for further protein separation and detection.

### Terminal deoxynucleotidyl transferase-mediated nick-end labeling (TUNEL) staining

Based on the manufacturer's protocol for the *in situ* Cell Death Detection Kit (11684795910, Roche, Mannheim, Germany), TUNEL staining was performed to detect cell death in brain sections. Six random fields from each group were observed using a fluorescence microscope (Olympus BX50/BX-FLA/DP70; Olympus). Image J software was used to analyze the positive rate of TUNEL staining by an observer who was blind to the experimental groups.

### Fluoro-jade B (FJB) staining

Neuronal degeneration in brain tissues was detected by FJB staining as described previously (Wang et al., 2017). Briefly, after being dewaxed, brain sections were incubated with 0.06% KMnO_4_ solution in the dark at room temperature for 15 min. The sections were then washed with PBS and incubated with FJB working solution (containing 0.1% acetic acid) for 60 min. Subsequently, the sections were washed and air-dried at room temperature in the dark. Finally, the sections were observed under a fluorescence microscope (Olympus BX50/BXFLA/DP70; Olympus). Image J software was used to analyze FJB staining. Quantitative analysis was performed by an observer who was blind to the experimental groups.

### Statistical analysis

Values are presented as mean ± SEM. GraphPad Prism 5 (GraphPad Software Inc., San Diego, CA, USA) was used for all statistical analysis. One-way ANOVA for multiple comparisons and a Student-Newman-Keuls *post-hoc* test were used to determine significant differences among all groups. A probability of *p* < 0.05 was considered statistically significant.

## Results

### ICH increased protein levels and secretion of netrin-1

**Figure 2:**
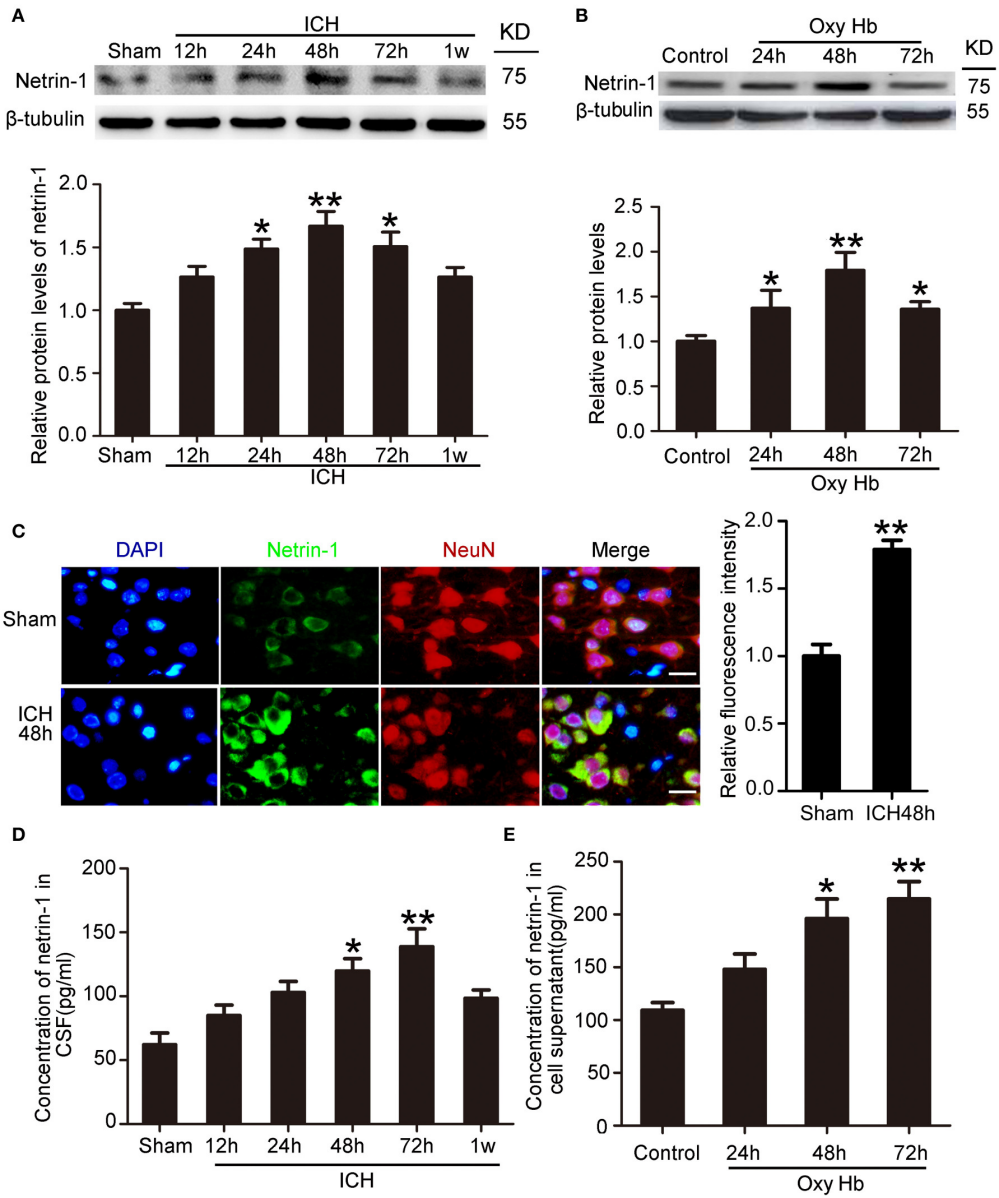
Changes in protein levels and secretion of netrin-1 after ICH. **(A)** Western blot analysis of netrin-1 in the peri-hematoma cortex at different time points after ICH. All values are expressed as mean ± SEM. Mean values of protein levels in the sham group were normalized to 1.0. ^*^p < 0.05, ^**^p < 0.01 vs. the sham group, *n* = 6. **(B)** Western blot of netrin-1 in cultured neurons exposed to OxyHb. All values are expressed as mean ± SEM. Mean values of protein levels in the control group were normalized to 1.0. ^*^p < 0.05, ^**^p < 0.01 vs. the control group, *n* = 3. **(C)** Double immunofluorescence staining was performed in the peri-hematoma cortex with netrin-1 antibody (green) and a neuronal marker (NeuN, red), and nuclei were labeled with DAPI (blue). Scale bar = 20 μm, ^**^p < 0.01 vs. the sham group, *n* = 6. **(D,E)** Concentrations of netrin-1 in CSF and cell supernatants were measured by ELISA. ^*^p < 0.05 and ^**^p < 0.01 vs. the sham group or control group (**D**, *n* = 6; **E**, *n* = 3).

To determine the potential role of netrin-1 in ICH-induced SBI, we first investigated protein levels and secretion of netrin-1 after ICH. *In vivo*, western blot analysis was performed 12, 24, 48, 72, and 1 week after the ICH model was established. Protein levels of netrin-1 in the peri-hematoma cortex increased by a factor of 1.7 in the ICH (48 h) group compared with the sham group (Figure 2A). *In vitro*, western blot analysis was performed on cultured neurons at 24, 48, and 72 h after exposure to OxyHb. The results demonstrated that protein levels of netrin-1 increased significantly 24 h after OxyHb treatment compared with the control group. Levels peaked at 48 h and then declined gradually (Figure 2B). In addition, immunofluorescence staining further showed an increase in protein levels of netrin-1 in neurons in the peri-hematoma cortex (Figure 2C). Finally, to observe secretion of netrin-1, CSF and culture media concentrations of netrin-1 were measured by ELISA analysis (Figures 2D,E). Compared with the sham group, concentrations of netrin-1 in CSF peaked at 72 h after ICH and showed a nearly 1.4-fold increase (Figure 2D). Concentrations of netrin-1 in culture media of neurons exposed to OxyHb showed a similar trend (Figure 2E).

### ICH increased protein levels of DCC and UNC5H2

**Figure 3:**
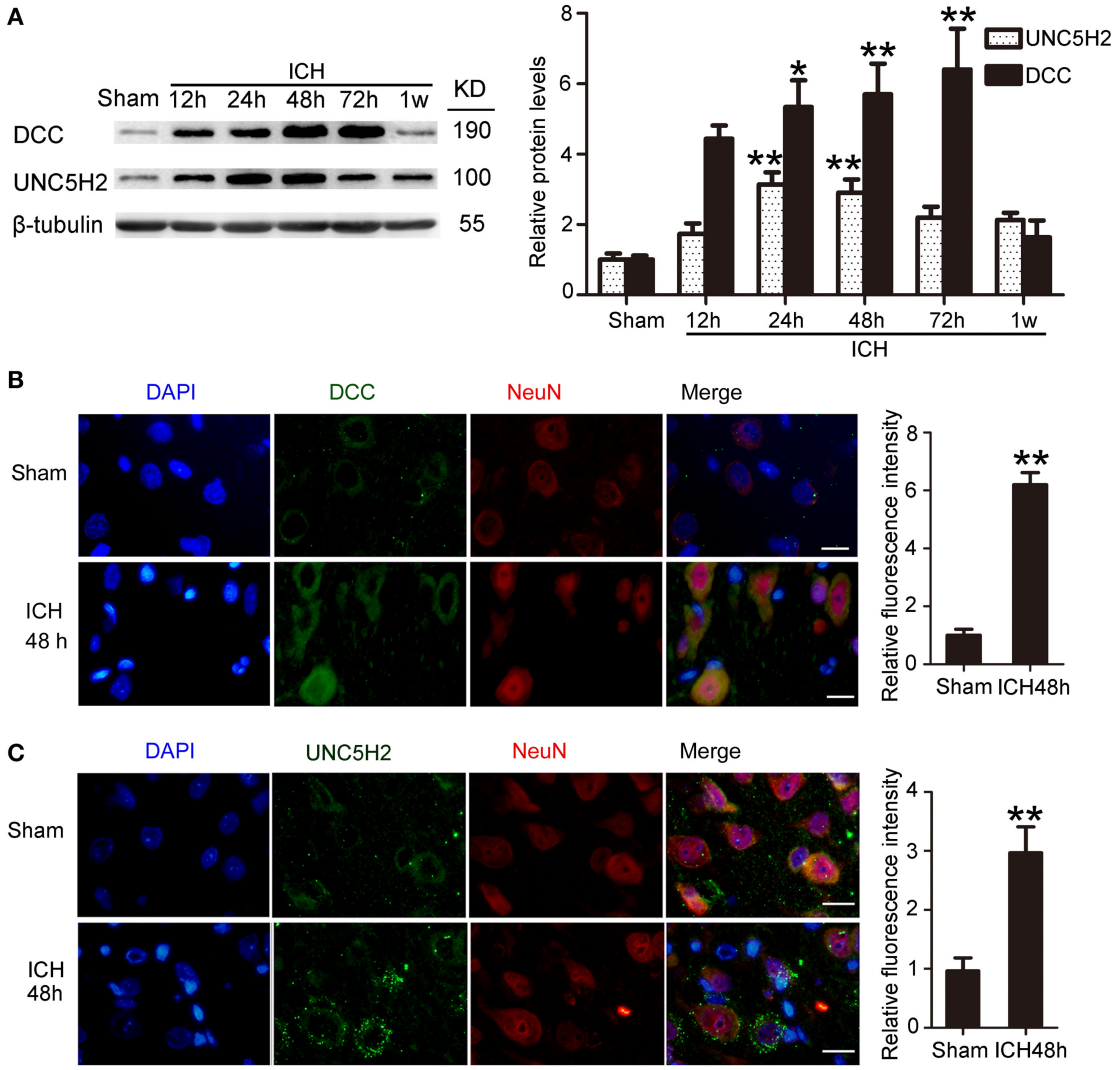
Protein levels and localization of DCC and UNC5H2 after ICH. **(A)** Western blot assay of protein levels of DCC and UNC5H2 in the peri-hematoma cortex at different time points after ICH. All values are expressed as mean ± SEM. Mean values of protein levels in the sham group were normalized to 1.0. ^*^p < 0.05, ^**^p < 0.01 vs. the sham group, *n* = 6. **(B)** Double immunofluorescence staining was performed in the peri-hematoma cortex with DCC antibody (green) and a neuronal marker (NeuN, red), and nuclei were labeled with DAPI (blue). Scale bar = 20 μm. All values are expressed as mean ± SEM. The mean value of the sham group was normalized to 1.0. ^**^p < 0.01 vs. the sham group, *n* = 6. **(C)** Double immunofluorescence staining was performed in the peri-hematoma cortex with UNC5H2 antibody (green) and a neuronal marker (NeuN, red), and nuclei were labeled with DAPI (blue). Scale bar = 20 μm. All values are expressed as mean ± SEM. The mean value of the sham group was normalized to 1.0. ^**^p < 0.01 vs. the sham group, *n* = 6.

Western blot was performed to assess protein levels of DCC and UNC5H2. Twenty-four hours after ICH, both DCC and UNC5H2 were upregulated in the peri-hematoma cortex, and DCC was elevated more significantly (Figure 3A). UNC5H2 reached the highest level with about a 3.6-fold enhancement during 24–48 h, while DCC peaked at 72 h and showed nearly a 6.2-fold increase. Immunofluorescence staining of DCC and UNC5H2 was also performed to assess further the upregulated protein levels in neurons in the peri-hematoma cortex. Consistent with their roles as membrane receptors, DCC and UNC5H2 were localized to the peripheral region of neurons (Figures 3B,C).

### Interactions between netrin-1 and its receptors after ICH

**Figure 4:**
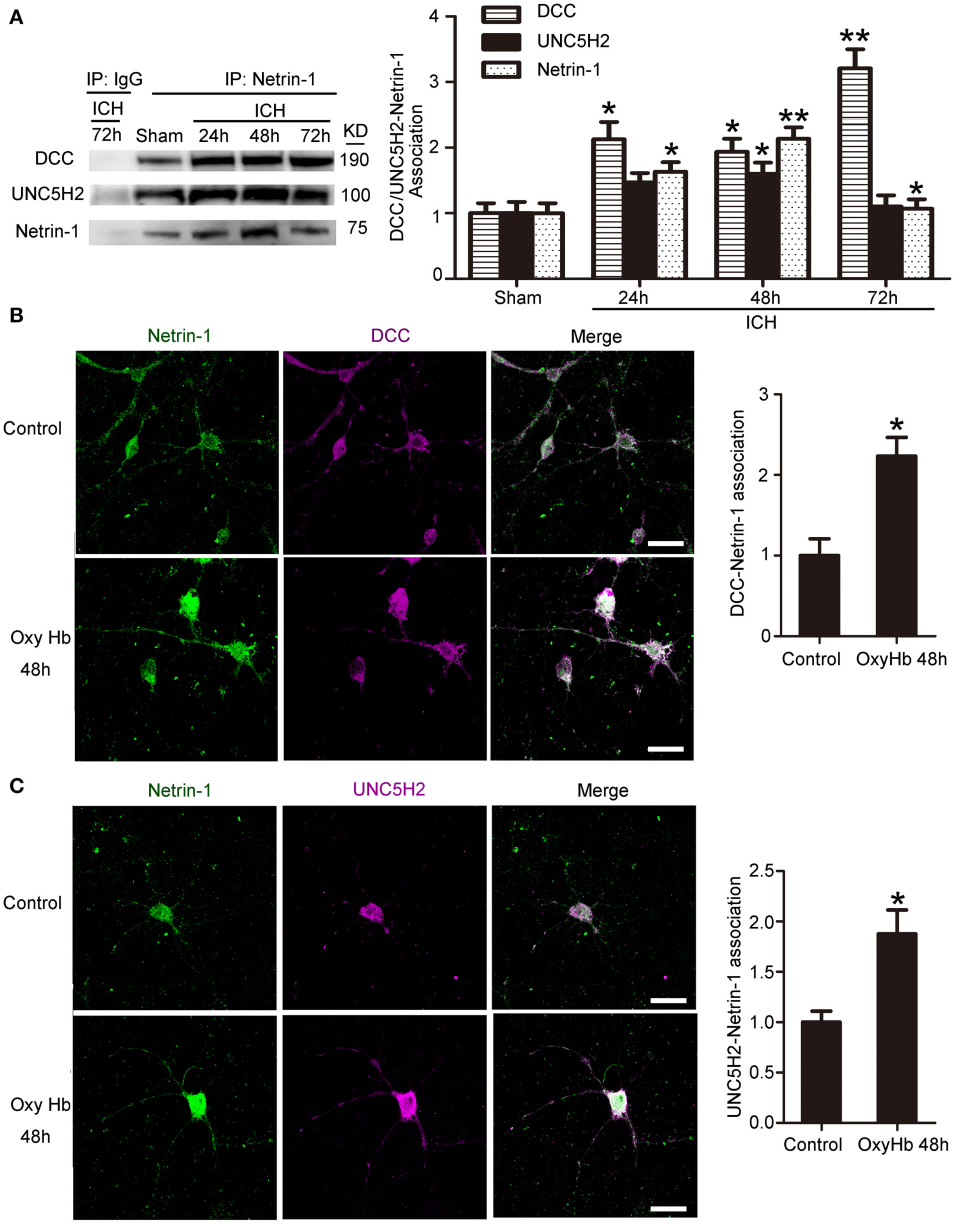
Interactions between netrin-1 and DCC/UNC5H2 after ICH. **(A)** Netrin-1/DCC and netrin-1/UNC5H2 interactions in the peri-hematoma cortex were determined by a co-immunoprecipitation assay. Quantitative histogram analysis was performed. All values are expressed as mean ± SEM. The mean value of the sham group was normalized to 1.0. ^*^p < 0.05, ^**^p < 0.01 vs. the sham group, *n* = 6. **(B)** Double immunofluorescence analysis was performed with netrin-1 antibody (green) and DCC antibody (purple) to observe localization and interactions. Scale bar = 20 μm. All values are expressed as mean ± SEM. The mean value of the sham group was normalized to 1.0. ^*^p < 0.05 vs. the control group, *n* = 3. **(C)** Double immunofluorescence analysis was performed with netrin-1 antibody (green) and UNC5H2 antibody (purple). Scale bar = 20 μm. All values are expressed as mean ± SEM. The mean value of the sham group was normalized to 1.0. ^*^p < 0.05 vs. the control group, *n* = 3.

Next, we investigated interactions between netrin-1 and DCC/UNC5H2 by CO-IP (Figure 4A). Levels of both DCC and UNC5H2 binding to netrin-1 significantly increased after ICH. However, the magnitude of the increase (a 3.2-fold increase for the DCC/netrin-1 interaction and a 1.6-fold increase for the UNC5H2/netrin-1 interaction) was much less than that for total protein as shown in Figure 3A. Therefore, the quantity of unbound DCC/UNC5H2 may increase after ICH. Double immunofluorescence analysis further verified an OxyHb-induced increase in interactions between netrin-1 and DCC/UNC5H2 in cultured neurons (Figures 4B,C).

### ICH decreased protein levels of KIF1A

**Figure 5:**
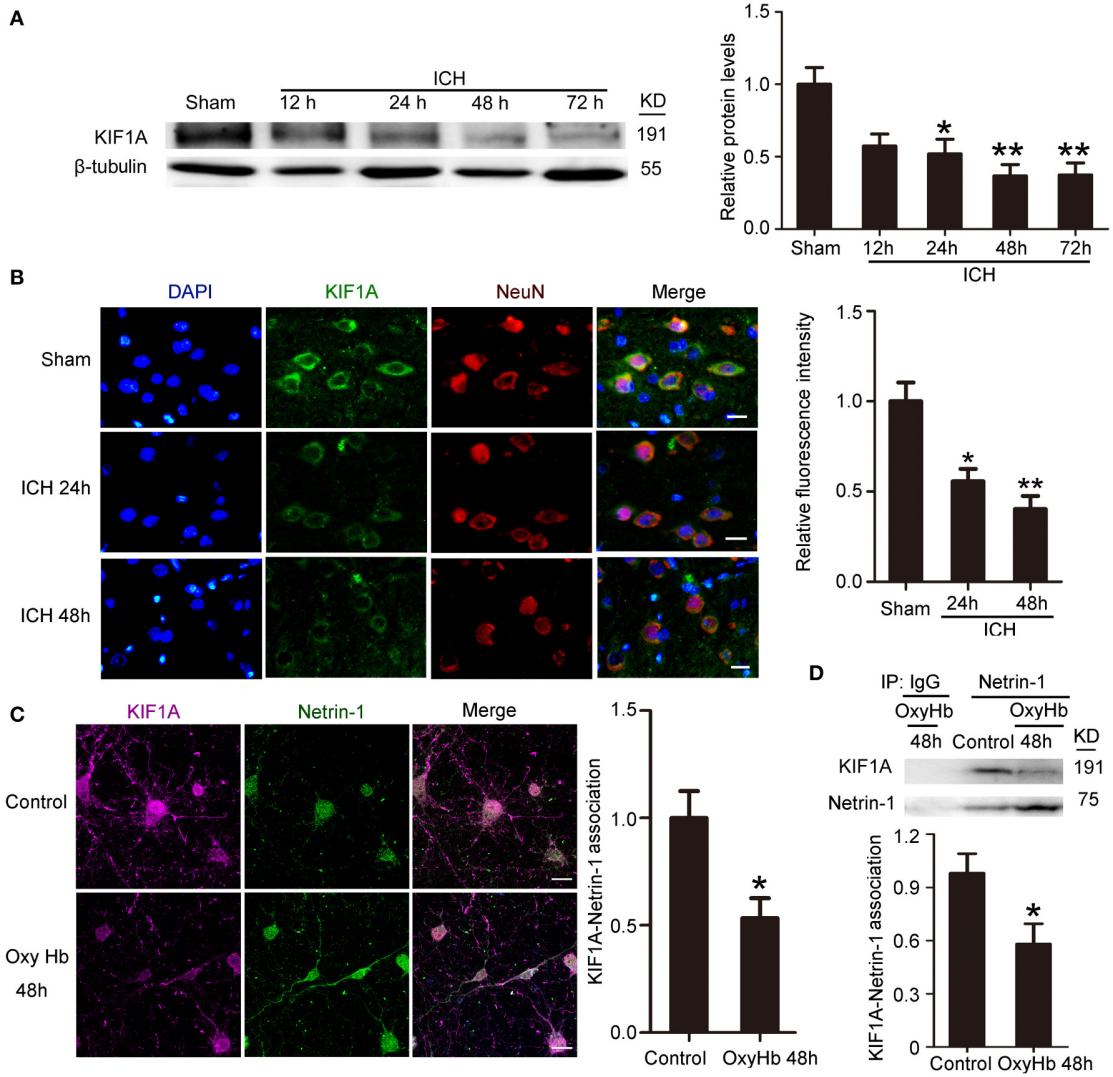
Changes in protein levels and localization of KIF1A after ICH**. (A)** Western blot assay of KIF1A protein levels in the peri-hematoma cortex at different time points after ICH. All values are expressed as mean ± SEM. Mean values of protein levels in the sham group were normalized to 1.0. ^*^p < 0.05 and ^**^p < 0.01 vs. the sham group, *n* = 6. **(B)** Double immunofluorescence staining was performed with KIF1A antibody (green) and a neuronal marker (NeuN, red), and nuclei were labeled with DAPI (blue). Scale bar = 20 μm. All values are expressed as mean ± SEM. The mean value of the sham group was normalized to 1.0. ^*^p < 0.05 and ^**^p < 0.01 vs. the sham group, *n* = 6. **(C)** Double immunofluorescence analysis was performed with netrin-1 antibody (green) and KIF1A antibody (purple). Scale bar = 20 μm. All values are expressed as mean ± SEM. The mean value of the control group was normalized to 1.0. ^*^p < 0.05 vs. the control group, *n* = 3. **(D)** KIF1A/netrin-1 interactions in the peri-hematoma cortex were assessed by a co-immunoprecipitation assay. Quantitative histogram analysis was performed. All values are expressed as mean ± SEM. ^*^p < 0.05 vs. the control group, *n* = 3.

To investigate further the role of KIF1A in ICH-induced SBI, we first performed a time course assay of KIF1A protein levels in the peri-hematoma cortex by western blot and immunofluorescence staining. Compared with the sham group, protein levels of KIF1A decreased significantly with time in the acute phase after induction of ICH and decreased to approximately one third of that in the sham group (Figure 5A). In addition, immunofluorescence assays further confirmed an ICH-induced decrease in protein levels of KIF1A in cortical neurons around the hematoma (Figure 5B). Furthermore, double immunofluorescence was performed *in vitro* to observe the subcellular localization of KIF1A and the interaction between KIF1A and netrin-1(Figure 5C). Similar to netrin-1, KIF1A was mainly observed in the cytoplasm and axons. Co-localization of KIF1A and netrin-1 decreased by about 40% in the OxyHb (48 h) group compared with the control group (Figure 5C). CO-IP further verified an ICH-induced decrease in the interaction between KIF1A and netrin-1 (Figure 5D). Thus, the ICH-induced increase in netrin-1 was accompanied by a decrease in KIF1A (Figures 2, 5).

### Exogenous recombinant netrin-1 exerted *in vivo* rescue effects on ICH-induced SBI

**Figure 6:**
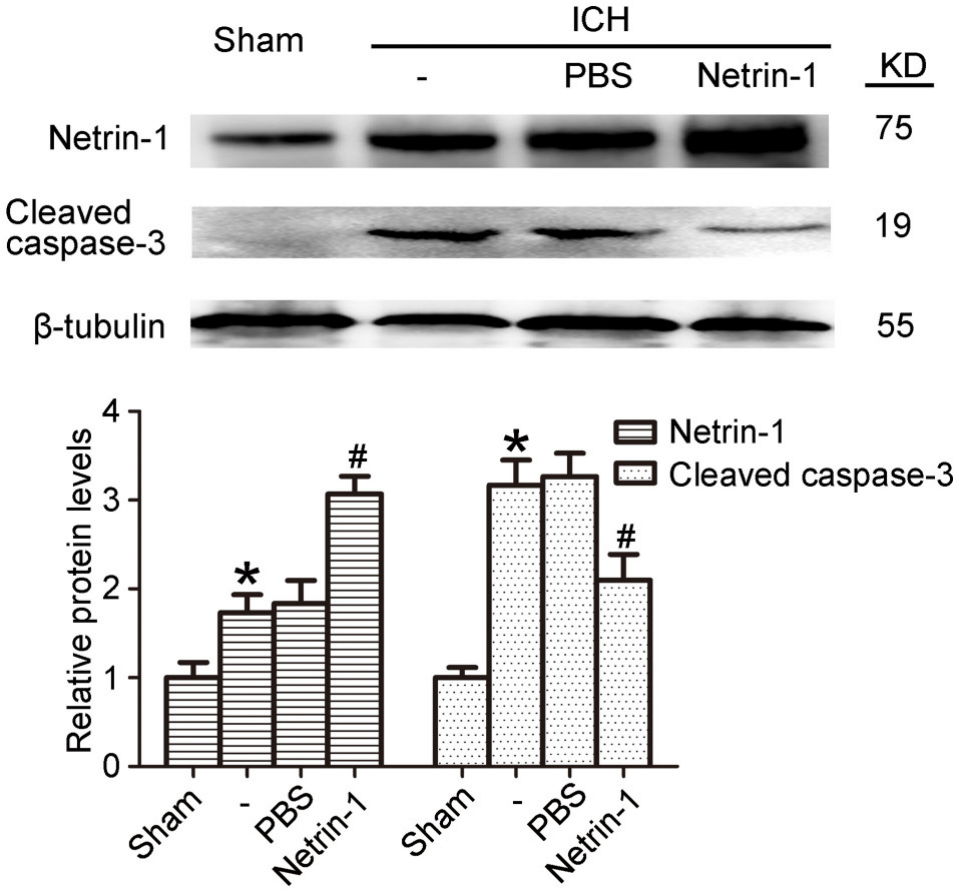
Effects of exogenous recombinant netrin-1 on ICH-induced SBI. Western blot of protein levels of netrin-1 and cleaved caspase-3 in the peri-hematoma cortex at 48 h after ICH onset. Mean values of protein levels in the sham group were normalized to 1.0. All values are expressed as mean ± SEM. ^*^p < 0.05 vs. the sham group. #*p* < 0.05 vs. the ICH + PBS group, *n* = 6.

**Figure 7:**
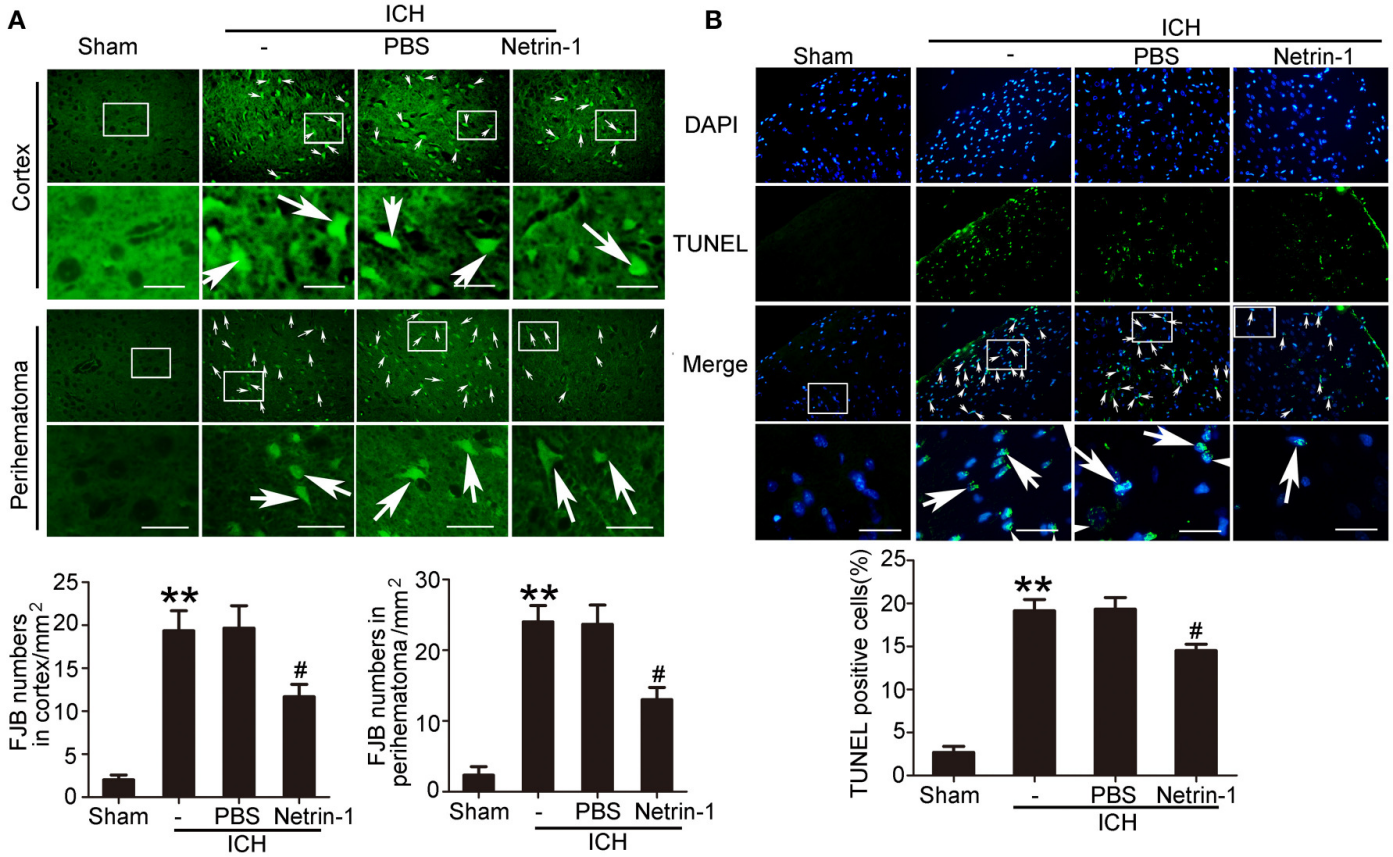
Effects of exogenous recombinant netrin-1 on ICH-induced SBI. **(A)** FJB staining (green) shows neuronal degradation in the cerebral cortex and peri-hematoma region. Scale bar = 32 μm. Arrows indicate FJB-positive cells, and number of FJB-positive cells/mm^2^ was determined. All values are expressed as mean ± SEM. ^**^p < 0.01 vs. the sham group. #*p* < 0.05 vs. the ICH + PBS group, *n* = 6. **(B)** TUNEL (green) with DAPI (blue) staining in the peri-hematoma cortex and determination of the percentage of TUNEL-positive cells. Arrows indicate apoptotic cells. Scale bar = 32 μm. All values are expressed as mean ± SEM. ^**^p < 0.01 vs. the sham group. #*p* < 0.05 vs. the ICH + PBS group, *n* = 6.

**Figure 8:**
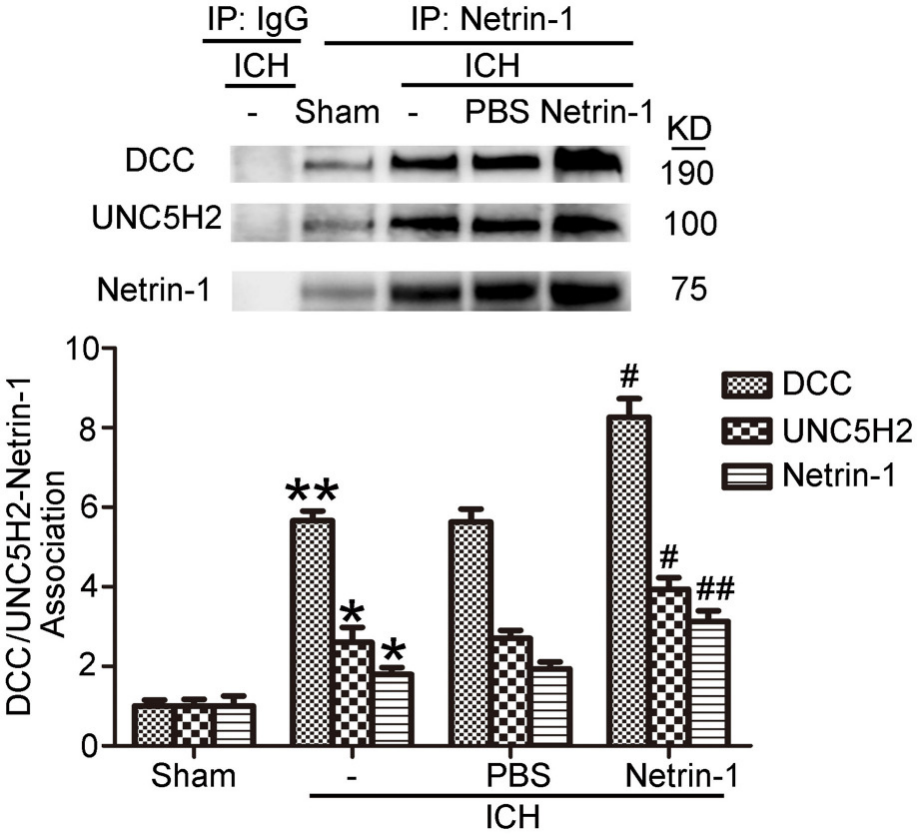
Effects of exogenous recombinant netrin-1 on interactions between netrin-1 and DCC/UNC5H2. Netrin-1/DCC and netrin-1/UNC5H2 interactions in the peri-hematoma cortex were assessed by a co-immunoprecipitation assay. All values are expressed as mean ± SEM. Mean values of protein levels in the sham group were normalized to 1.0. ^*^p < 0.05, ^**^p < 0.01 vs. the sham group. #*p* < 0.05, ##*p* < 0.01 vs. the ICH + PBS group, *n* = 6.

Intraventricular injection of exogenous recombinant netrin-1 was performed, and its effects on brain content of netrin-1 were assessed by western blot assay (Figure 6). Caspase-3 is a member of the cysteine-aspartic acid protease (caspase) family, and sequential cleavage of caspase-3 as its activation play a dominant role in the execution-phase of cell apoptosis (Perry et al., 1997). We found that ICH caused a significant increase in caspase-3 activation, and this effect was inhibited by netrin-1 injection (Figure 6). FJB staining was performed to evaluate neuronal degeneration and showed that netrin-1 administration significantly reduced the number of FJB-positive cells in both the cortex and peri-hematoma regions of ICH rats (Figure 7A). Next, brain cell death was observed by TUNEL staining (Figure 7B). The results showed that netrin-1 treatment decreased the number of TUNEL-positive neurons to 70% of that found in the PBS-injected groups. These findings indicate remarkable rescue effects of exogenous recombinant netrin-1 on ICH-induced neuronal degradation and death. To understand the mechanisms underlying neuroprotective effects of netrin-1, we analyzed the effects of netrin-1 intraventricular injection on the interactions between netrin-1 and its receptors by CO-IP (Figure 8). When netrin-1 content in the brain was upregulated by exogenous recombinant netrin-1 treatment, levels of DCC and UNC5H2 binding to netrin-1 increased, which suggests that levels of unliganded DCC and UNC5H2 may have been reduced.

### KIF1A promoted netrin-1 secretion

**Figure 9:**
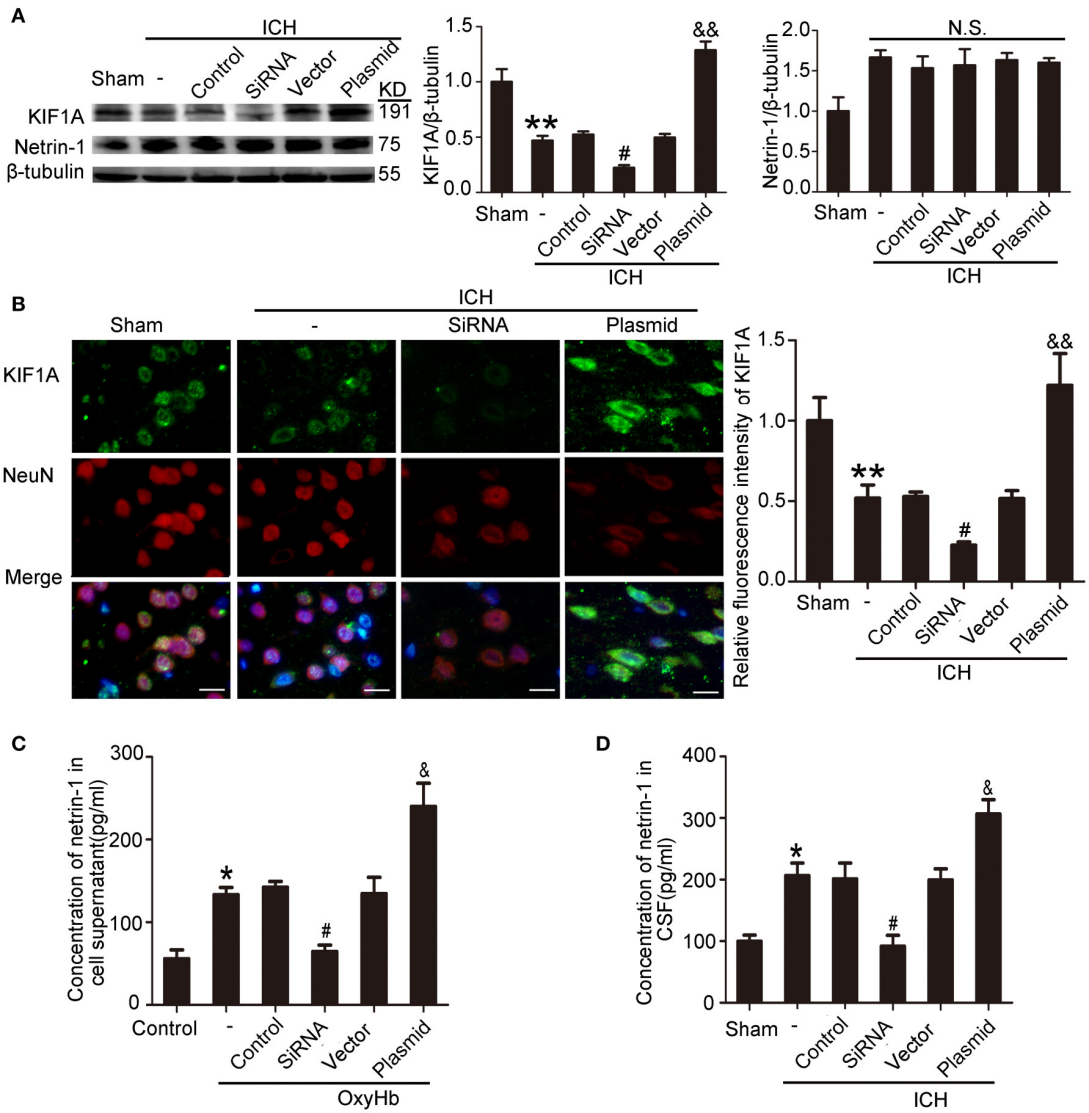
Effects of KIF1A interventions on expression of KIF1A and netrin-1 after ICH-induced SBI. **(A)** Western blot analysis of KIF1A and netrin-1 protein levels in the peri-hematoma cortex. All values are expressed as mean ± SEM. Mean values of protein levels in the sham group were normalized to 1.0. ^**^p < 0.01 vs. the sham group, #*p* < 0.05 vs. the ICH + control siRNA group, &&*p* < 0.01 vs. the ICH + vector group. N.S: no significant differences, *n* = 6. **(B)** Double immunofluorescence analysis was performed with KIF1A antibody (green) and NeuN antibody (red). Scale bar = 20 μm. All values are expressed as mean ± SEM. The mean value of the sham group was normalized to 1.0. ^**^p < 0.01 vs. the sham group, #*p* < 0.05 vs. the ICH + control siRNA group, &&*p* < 0.01 vs. the ICH + vector group, *n* = 6. **(C,D)** Concentrations of netrin-1 in cell supernatants and CSF were measured by ELISA. All values are expressed as mean ± SEM. ^*^p < 0.05 vs. the sham or control group, #*p* < 0.05 vs. the ICH + control siRNA or OxyHb + control siRNA group, &*p* < 0.05 vs. the ICH + vector or OxyHb + vector group (**C**, *n* = 3; **D**, *n* = 6).

To evaluate the potential role of KIF1A in netrin-1 secretion, knockdown, and overexpression of KIF1A was performed, and the effects were evaluated by western blot and immunofluorescent labeling (Figures 9A,B). Although total protein level of netrin-1 were not affected by KIF1A knockdown and overexpression (Figure 9A), ELISA assay showed that concentrations of netrin-1 in both CSF and cell supernatants increased following upregulation of KIF1A and decreased following downregulation of KIF1A, which suggests that KIF1A likely participates in netrin-1 secretion (Figures 9C,D).

### KIF1A inhibited ICH-induced SBI

**Figure 10:**
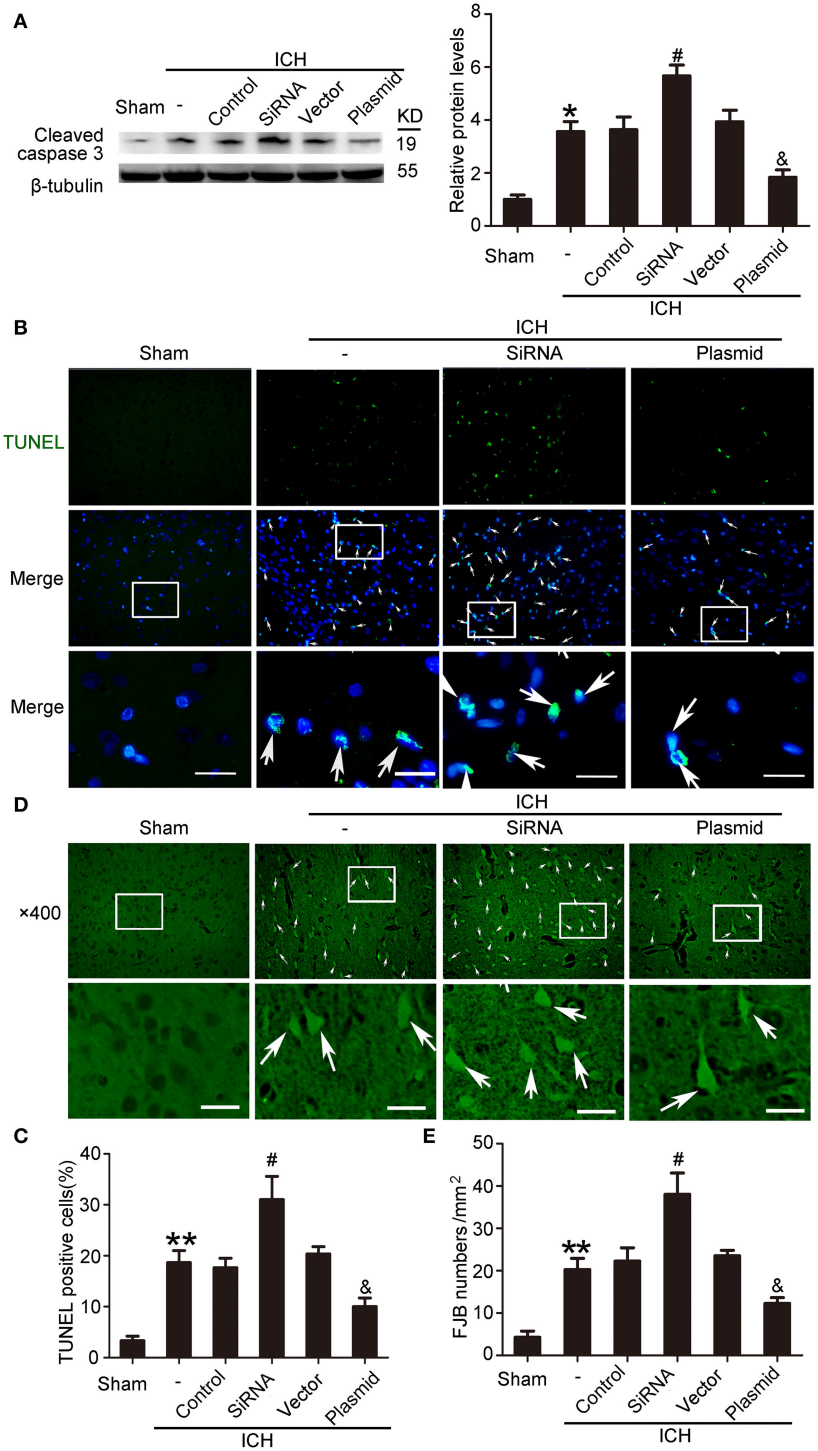
Effects of KIF1A intervention on ICH-induced SBI. **(A)** Western blot of protein levels of cleaved caspase-3. All values are expressed as mean ± SEM. Mean values of protein levels in the sham group were normalized to 1.0. ^*^p < 0.05 vs. the sham group, #*p* < 0.05 vs. the ICH + control siRNA group, &*p* < 0.05 vs. the ICH + vector group, *n* = 6. **(B)** TUNEL (green) with DAPI (blue) staining in the peri-hematoma cortex. Arrows indicate TUNEL-positive cells. Scale bar = 32 μm. **(C)** Determination of the percentage of TUNEL-positive cells. All values are expressed as mean ± SEM. ^**^p < 0.01 vs. the sham group, #*p* < 0.05 vs. the ICH + control siRNA group, &*p* < 0.05 vs. the ICH + vector group, *n* = 6. **(D)** FJB staining (green) shows neuronal degradation in the peri-hematoma cortex. Scale bar = 32 μm. Arrows indicate FJB-positive cells. **(E)** Number of FJB-positive cells/mm^2^ was determined. All values are expressed as mean ± SEM. ^**^p < 0.01 vs. the sham group, #*p* < 0.05 vs. the ICH + control siRNA group, &*p* < 0.05 vs. the ICH + vector group, *n* = 6.

Western blot assays showed that, compared with the ICH + control siRNA group, levels of cleaved caspase-3 significantly increased in the ICH + KIF1A-siRNA group in the peri-hematoma cortex. In contrast, overexpression of KIF1A reduced protein levels of cleaved caspase-3 in the peri-hematoma cortex compared with the rats treated with vector (Figure 10A). In addition, KIF1A overexpression reduced the number of TUNEL-positive cells, and KIF1A knockdown had the opposite effect (Figures 10B,C). Similarly, KIF1A overexpression significantly decreased the number of FJB-positive cells, which increased following siRNA intervention (Figures 10D,E). These results suggest that upregulation of KIF1A can inhibit ICH-induced neuronal degeneration and death in the brain.

**Figure 11:**
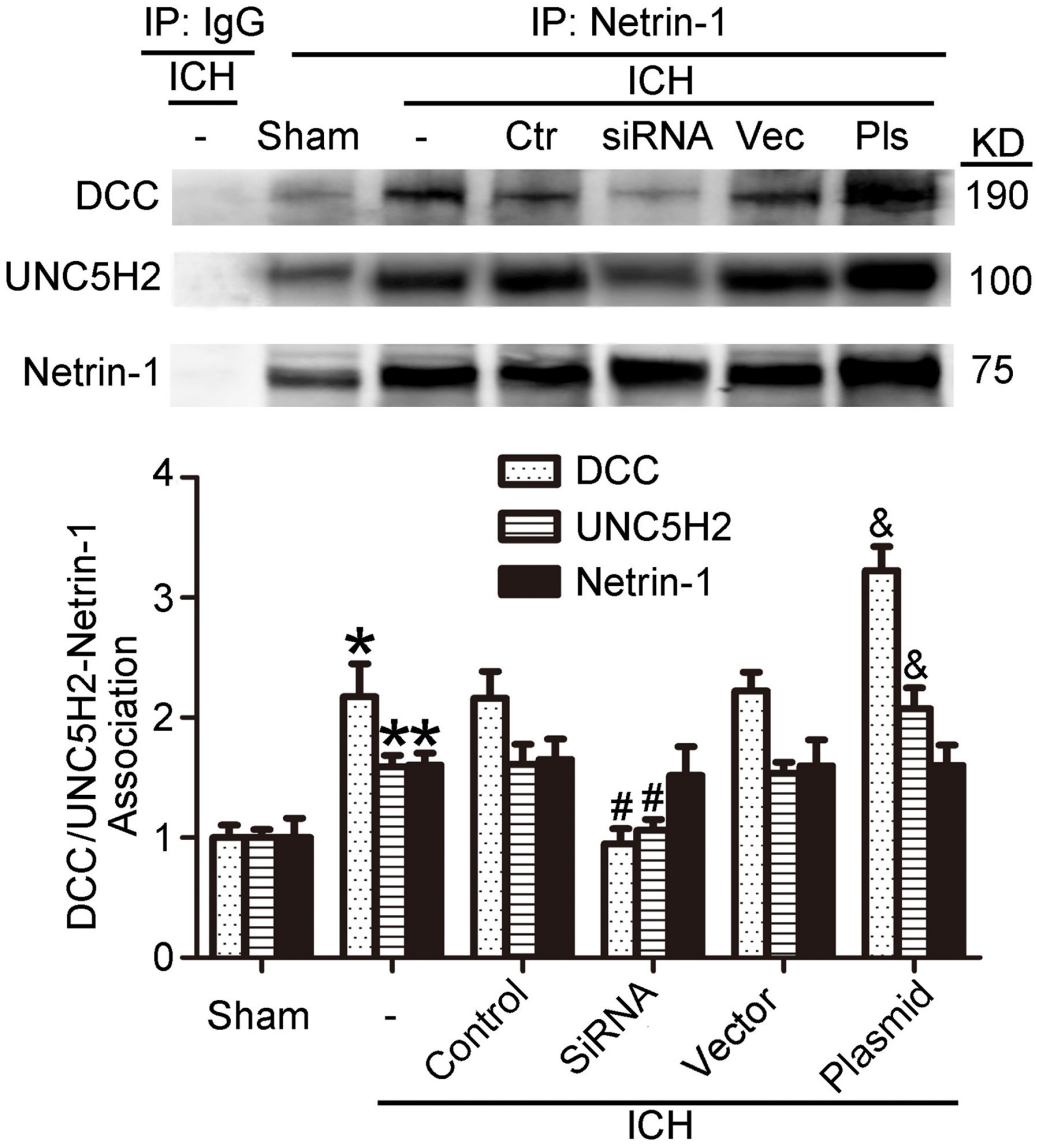
Effects of KIF1A intervention on interactions between netrin-1 and DCC/ UNC5H2. Netrin-1/DCC and netrin-1/UNC5H2 interactions in the peri-hematoma cortex were assessed by a co-immunoprecipitation assay. All values are expressed as mean ± SEM. Mean values of protein levels in the sham group were normalized to 1.0. ^*^p < 0.05 vs. the sham group, #*p* < 0.05 vs. the ICH + control siRNA group, &*p* < 0.05 vs. the ICH + vector group, *n* = 6.

As shown in Figures 9C,D, kinesin motor KIF1A overexpression elevated netrin-1 secretion. To identify further the potential mechanisms underlying the protective effects of KIF1A against brain injury following ICH, CO-IP was performed to assess interactions between netrin-1 and its receptors. The KIF1A overexpression group showed a significant increase in interactions between netrin-1 and DCC/UNC5H2 compared with the vector group. In contrast, the siRNA group showed lower interaction levels compared with the control siRNA group (Figure 11). These data further indicate that the kinesin motor protein, KIF1A, may be involved in intracellular transport and secretion of netrin-1.

## Discussion

As reported previously, netrin-1 binds to DCC and generally UNC5H1, H2, H3, and H4 in rats or UNC5A, B, C, and D in humans (Guenebeaud et al., 2010; Webber et al., 2011). Netrin-1/receptor complexes have been shown to be involved in cell migration regulation and adhesion in development of the nervous system, muscle, vasculature, lung, pancreas, and mammary glands (Llambi et al., 2001). More recently, roles of netrin-1 in adult and postnatal development have been investigated. Interestingly, its receptors also act as dependence receptors (Mehlen and Tauszig-Delamasure, 2014). In the absence of netrin-1, DCC, and UNC5H2 trigger apoptotic signals, and this process can be reversed by binding netrin-1. However, whether loss of netrin-1 promotes cell death is controversial (Williams et al., 2006; Furne et al., 2008). Bin et al. showed that netrin-1-deficient mice die earlier and exhibit severe axon guidance defects. However, an increase in the number of dying cells was not detected, and these findings are inconsistent with netrin-1 as an essential dependence receptor ligand in the embryonic spinal cord (Bin et al., 2015). Although it is controversial, most reports suggest protective effects of netrin-1 and support the dependence-receptor hypothesis. Thus, we designed this study based on the proapoptotic dependence-receptor hypothesis, in which a dependence receptor activates apoptosis in the absence of a ligand.

KIF1A is a type of molecular motor in neurons and is the major axonal transporter of synaptic vesicles (Hung and Coleman, 2016; Zhang et al., 2016). In general, KIF1A recognizes and binds to its cargo in the neuron cell body and then transports the cargo to synapses, where it is released (Fuchs and Westermann, 2005; Goldstein et al., 2008). It has been demonstrated that KIF1A is degraded upon release of its cargo in synaptic regions. Thus, cargo binding or cargo release may be possible mechanisms for regulating KIF1A protein levels (Kumar et al., 2010). In addition, LIN-2 (CASK) was reported as a regulator of KIF1A clustering and motility in neurons (Wu et al., 2016). In recent years, KIF1A has been more widely investigated in the nervous system (Ohba et al., 2015; Hung and Coleman, 2016; Tanaka et al., 2016; Krenn et al., 2017). In 2016, we found 19 reports that included KIF1A, and most involved studying neurons. KIF1A has been shown to be essential for sensory neuron survival and function via transport of the TrkA neurotrophin receptor (Tanaka et al., 2016). In addition, KIF1A was demonstrated that it is related to neurodevelopment and autophagy at synapses, site-specific synapse maturation, as well as size and density regulation of synapses (Niwa et al., 2016; Stavoe et al., 2016; Zhang et al., 2016). However, there have been no previous reports on the role of KIF1A in ICH-induced SBI. This study is the first to demonstrate the rescue effects of KIF1A overexpression in ICH-induced SBI.

**Figure 12:**
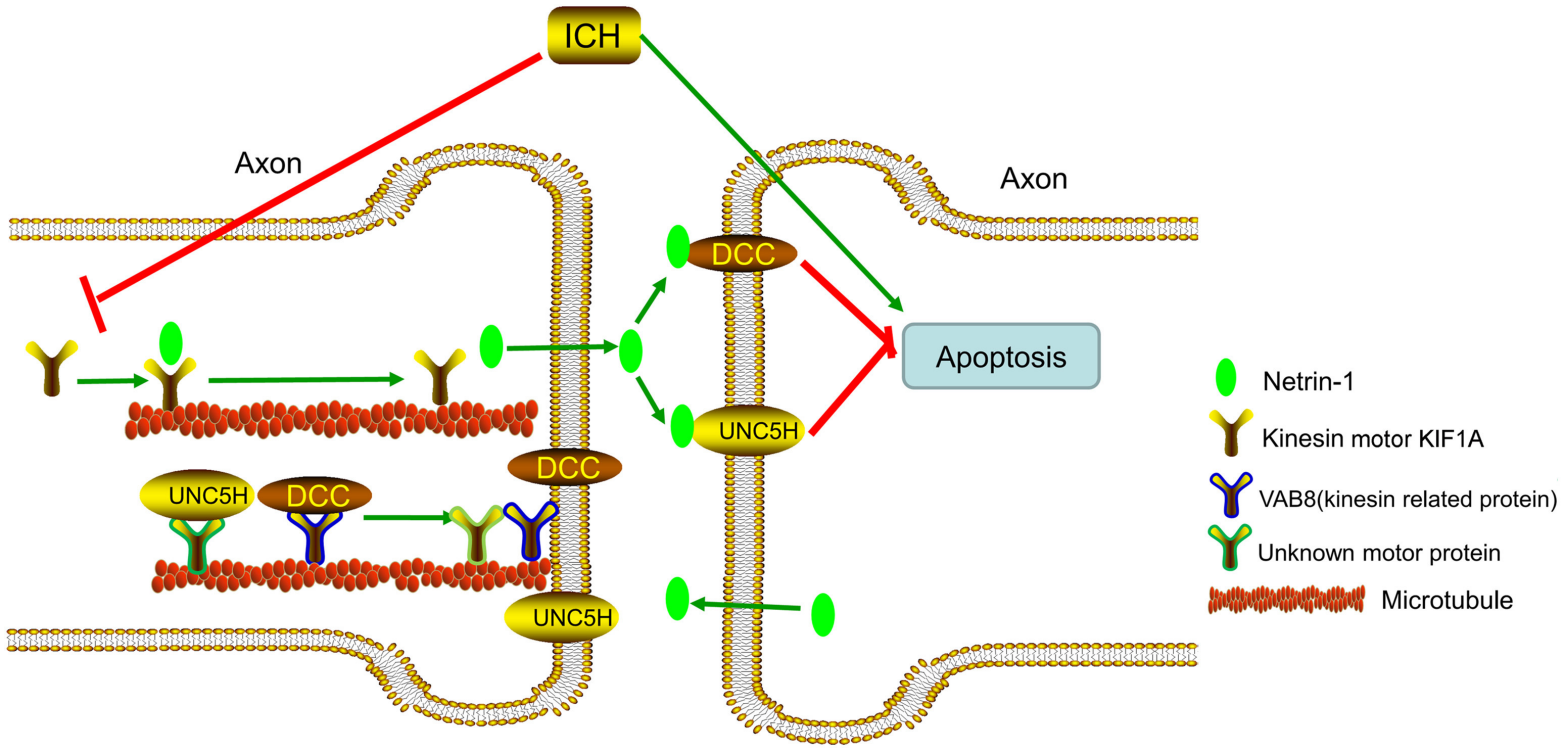
Model illustrating possible mechanisms underlying the roles of kinesin motor KIF1A, netrin-1, and their interactions in SBI after ICH. The kinesin motor, KIF1A, serves as a transporter during netrin-1 secretion. KIF1A binds to netrin-1 and transports it to the axon. When ICH occurs, KIF1A is downregulated, which inhibits intracellular transport and secretion of netrin-1. This leads to a decrease in binding of extracellular netrin-1 to “dependence receptors,” DCC and UNC5H2. The increase in number of unliganded receptors triggers apoptotic signal transduction and subsequent cell death. This pathological process aggravates SBI after ICH. Therefore, upregulation of KIF1A or administration of exogenous netrin-1 may inhibit ICH-induced SBI.

KIF1A serves as a transporter in netrin-1 secretion (Ogura et al., 2012). When ICH occurs, protein levels of KIF1A decrease, which decreases secretion of intracellular netrin-1 into the extracellular space. As a result, extracellular netrin-1 binding to the dependence receptors, DCC and UNC5H2, decreases. The unliganded receptors then trigger apoptotic signal transduction, which leads to cell death. This pathological process aggravates SBI after ICH (Figure 12). When we upregulated KIF1A levels or administered exogenous netrin-1, ICH-induced SBI was inhibited. In addition, as shown in Figure 9A, both knockdown and overexpression of KIF1A did not affect protein levels of netrin-1, which suggests that KIF1A exerts its neuroprotection by regulating netrin-1 secretion rather than by altering levels of netrin-1 protein.

When ICH occurs, a decrease in KIF1A (Figure 5A) would subsequently inhibit netrin-1 secretion. However, as shown in Figure 2D, the concentration of netrin-1 in CSF reached about 120 pg/mL at 48 h and peaked at 72 h after ICH. The increase in netrin-1 secretion may be induced by an increase in protein levels of netrin-1 after ICH (Figure 2A) since KIF1A transport is not saturated. In addition, the increases in protein levels of DCC and UNC5H2 (Figure 3A) were much higher than the complexes of DCC/netrin-1 and UNC5H2/netrin-1 at 48 h after ICH (Figure 4A), which suggests that endogenous netrin-1 secretion was insufficient to downregulate DCC- and UNC5H2-mediated apoptotic signals and subsequent SBI. In addition, KIF1A overexpression led to a nearly 3.1-fold increase of netrin-1 secretion, while concentration of netrin-1 in CSF reached about 300 pg/mL at 48 h after ICH (Figure 9C). Rescue by exogenous recombinant netrin-1 (Figures 6, 7) and KIF1A overexpression (Figure 10) further suggest that increases in protein levels of netrin-1 may be a neuroprotective strategy after ICH. However, this protective mechanism is significantly attenuated by ICH-induced loss of KIF1A.

As shown in Figure 2, both expression and secretion of netrin-1 were enhanced after ICH. Furthermore, compared with the sham group, protein levels of cleaved caspase-3 were shown to be increased in the ICH group and decreased after exogenous netrin-1 injection (Figure 6). These results suggest that ICH-induced enhancement of protein levels and secretion of netrin-1 is an endogenous neuroprotective mechanism. However, the enhancement is insufficient to neutralize ICH-induced UNC5H2/DCC-dependent apoptosis and, thus fails to inhibit subsequent brain cell death and SBI.

Finally, this study had limitations. The animals were intraventricularly injected with netrin-1, KIF1A-specific siRNA, and an expression plasmid for KIF1A. When these reagents crossed the ependyma and reached brain tissues, the effective amount and their biological activity could not be controlled accurately. In addition, we used only healthy adult rats to mimic the progression of human ICH. However, in clinical work, most ICH patients are older patients, and most suffer from hypertension. Furthermore, it was previously demonstrated that overexpression of netrin-1 can promote neovascularization in the adult brain *in vivo* and regulate the blood-brain barrier (Fan et al., 2008; Wen et al., 2014), which suggests that netrin-1 may play various roles in neuroprotection after ICH. Thus, it is not clear whether the dependence-receptor hypothesis, on which our experiments were based, constitutes the underlying mechanism. Finally, this study was designed and performed in rats, and clinical translation as well as therapeutic prospects remain to be confirmed.

In conclusion, the present study demonstrated that, in the early phase of ICH in a rat model, expression of netrin-1 and its dependence receptors DCC and UNC5H2 increased significantly. However, the overall result indicates an increase in the quantity of dependence receptors that are not bound to netrin-1. As a result, when netrin-1 was administered, neuronal death and degeneration in the early phase of ICH could be inhibited. In addition, it has been demonstrated previously that the kinesin motor protein, KIF1A, is involved in transport of netrin-1. KIF1A overexpression acts a suppressor of brain cell death and degeneration by accelerating netrin-1 transport and secretion.

## Author contributions

GC and CL conceived and designed the study, including quality assurance and control. JW, WZ, and ZY performed the experiments and wrote the paper. LS and HL designed the study's analytic strategy. HS and XL helped conduct the literature review and prepare the Materials and Methods section of the text. All authors read and approved the manuscript.

### Conflict of interest statement

The authors declare that the research was conducted in the absence of any commercial or financial relationships that could be construed as a potential conflict of interest.
